# Rhabdomyolysis secondary to systemic lupus erythematosus

**DOI:** 10.1007/s13317-018-0105-1

**Published:** 2018-04-06

**Authors:** Daniel Nguyen, Farah Alsaati, Jena Deitrick, Kamel Azhar, Evelyn Sbar

**Affiliations:** Texas Tech University Health Sciences Center Amarillo School of Medicine, 1400 S Coulter St, Amarillo, TX 79106 USA

**Keywords:** Systemic lupus erythematous, Rhabdomyolysis, Lupus, Myopathy

## Abstract

Systemic lupus erythematous (SLE) is a systemic auto-immune disorder with a variety of presentations and wide spread organ involvement. We present a case report of a patient with an SLE exacerbation as well as concurrent rhabdomyolysis with massively elevated CPK (304,700 U/L). Though a rarely reported effect of SLE, rhabdomyolysis can be severe and potentially lethal secondary or concurrent to an acute SLE episode. This case report demonstrates the association between SLE and rhabdomyolysis, which is not well described in the current literature.

## Introduction

Systemic lupus erythematosus (SLE) is an auto-immune disorder defined by the 2012 SLICC (Systemic Lupus International Collaborating Clinics Classification Criteria) criteria as having 4 or more out of 11 ARC (American College of Rheumatology) SLE criteria (with at least 1 clinical and 1 laboratory criteria) or a proven biopsy confirming lupus nephritis with a positive ANA or anti-dsDNA. We present a case report of a patient with evidence of both acute cutaneous and systemic lupus, along with synovitis involving two or more joints, positive ANA, and low complement levels of C3. In addition, this patient presented with severely elevated CPK values combined with myalgias suggestive of rhabdomyolysis. While drugs, infections, and inflammatory disorders have been described as causes of non-exertional rhabdomyolysis, SLE appears to be the main causative agent in this case.

## Case report

**Fig. 1:**
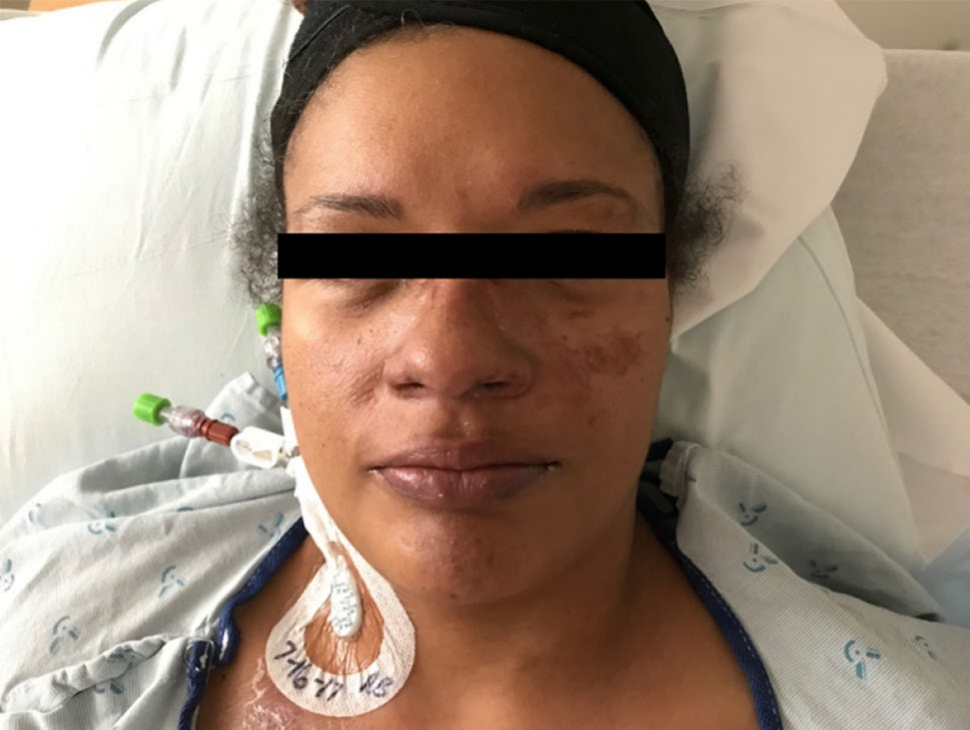
Malar rash that is purple/red in color that covers the cheeks and nose sparing the rest of the face

**Fig. 2:**
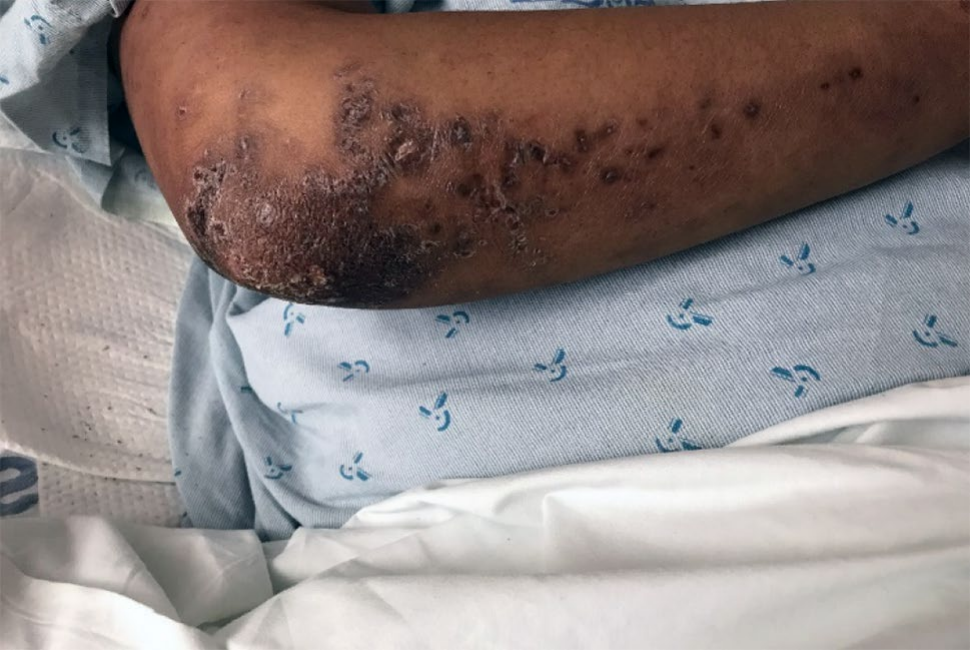
Subcutaneous rash that is erythematous and nodular

A 36-year-old African-American woman presented to the ER with a 3-day history of vomiting, diarrhea, full-body arthralgias, myalgias, and bilateral TMJ stiffness and tenderness, along with severe persistent headache. She had been seen in urgent care 1 week prior to admission with complaints of fever, nausea, and diarrhea. Her medical history was significant for rheumatoid arthritis that had been in remission for 12 years. She was not currently on any maintenance therapy for this auto-immune disease. On arrival, vitals were recorded as BP of 125/89, HR of 92 bpm, RR of 18/min, Temperature of 98.3 °F, and 100% SpO_2_ on room air. Physical exam was significant for a malar rash, shown in Fig. 1, as well as a second subcutaneous rash on her arms that was erythematous and nodular seen in Fig. 2. In addition, she had bilateral knee effusions along with weakness and edema of the lower extremities.

Labs obtained in the emergency room showed an elevated creatinine level of 2.85 mg/dL (0.5–1.0 mg/dL), AST 1293 U/L (10–40 U/L), ALT 397 U/L (7–56 U/L), elevated phosphorus 8.3 mg/dL (2.0–4.5 mg/dL), and a low potassium level of 3.1 mmol/L (3.5–5.0 mmol/L). CPK was extremely high at 6270 U/L (22–198 U/L) and CRP at 9.244 mg/L (< 3.0 mg/L). The initial differential diagnosis included dermatomyositis/polymyositis/SLE with rhabdomyolysis. She was admitted to the medical floor and given a 3 L bolus of normal saline along with IV methylprednisolone.

Despite the initial treatment with fluid and steroids, the patient’s creatinine and lab values worsened over the next 24 h. Her creatinine rose to 3.59 mg/dL, BUN increased to 70 mg/dL (7–20 mg/dL), and phosphate jumped to 9.8 mg/dL. In addition, CPK elevated to 207,300 U/L along with an increase in AST and ALT to 1797 and 482 U/L, respectively. Clinically, the patient was also deteriorating; she had been anuric since admission despite fluid resuscitation. The patient was transferred to the ICU, and nephrology and rheumatology were consulted. She was started on a bolus of intravenous corticosteroids to treat rhabdomyolysis secondary to polymyositis after consultants’ review. The rheumatologist ordered a panel of auto-immune markers to include CRP, RF, ANA, dsDNA, Ribosomal P, SSA (anti-Ro), SSB (anti-La), snRNP, anti-smith, RNP, centromere B, SCL-70, and Jo-1. The results revealed that the CRP was 5.556 mg/L (< 3.0 mg/L); the RF was 121 IU/mL (< 15 IU/mL), ANA was greater than 8 IU/mL (< 8 IU/mL), DsDNA was 1 IU/mL (0 IU/mL), Ribosomal P was greater than 8 IU/mL (IU/mL < 8), SSA (anti-Ro) was greater than 8 IU/mL (< 8 IU/mL), SSB (anti-La) was less than 0.2 IU/mL (< 1 IU/mL), snRNP was less than 0.2 IU/mL (< 1 IU/mL), anti-smith was less than 0.2 IU/mL (< 1 IU/mL), RNP was 4.4 IU/mL (< 1 IU/mL), centromere B was less than 0.2 IU/mL (< 1 IU/mL), SCL-70 was less than 0.2 IU/mL (< 1 IU/mL), and Jo-1 was less than 0.2 IU/mL (< 1 IU/mL).

These lab values suggested a probable diagnosis of SLE. Diagnostic criteria for SLE are based on ACR (American College of Rheumatology) guidelines with a sensitivity of 85% and specificity of 95% in patients with 4 out of 11 criteria. This patient had arthritis, photosensitivity, renal involvement, blood disorders, elevated ANA, immunologic phenomena with anti-dsDNA, and a malar rash, giving her an ACR SLE score of 7/11, defining her as having SLE. The SELENA–SLEDAI scoring matrix is a way to assess SLE disease activity using 24 different disease descriptors. Scores of six or above on the SLEDAI grid are considered to be consistent with active disease requiring therapy. The patient had a lupus headache, arthritis, myositis, urinary casts, a rash, low complement levels, and leukopenia having a total of 25 points on the SELENA–SLEDAI scoring matrix. This was consistent with a significant degree of active SLE, and need for aggressive medical therapy.

A central line was placed to initiate hemodialysis to alleviate her acute kidney injury. Prompt hemodialysis corrected her electrolytes and improved her creatinine and BUN within 24 h. However, her CPK continue to escalate to 304,700 U/L and her anuria persisted. The intravenous corticosteroids were tapered and changed to oral preparations to prevent further fluid overload and edema. Hydroxychloroquine was started 1 week after ruling out G6PD deficiency and the risk of iatrogenic hemolysis.

**Fig. 3:**
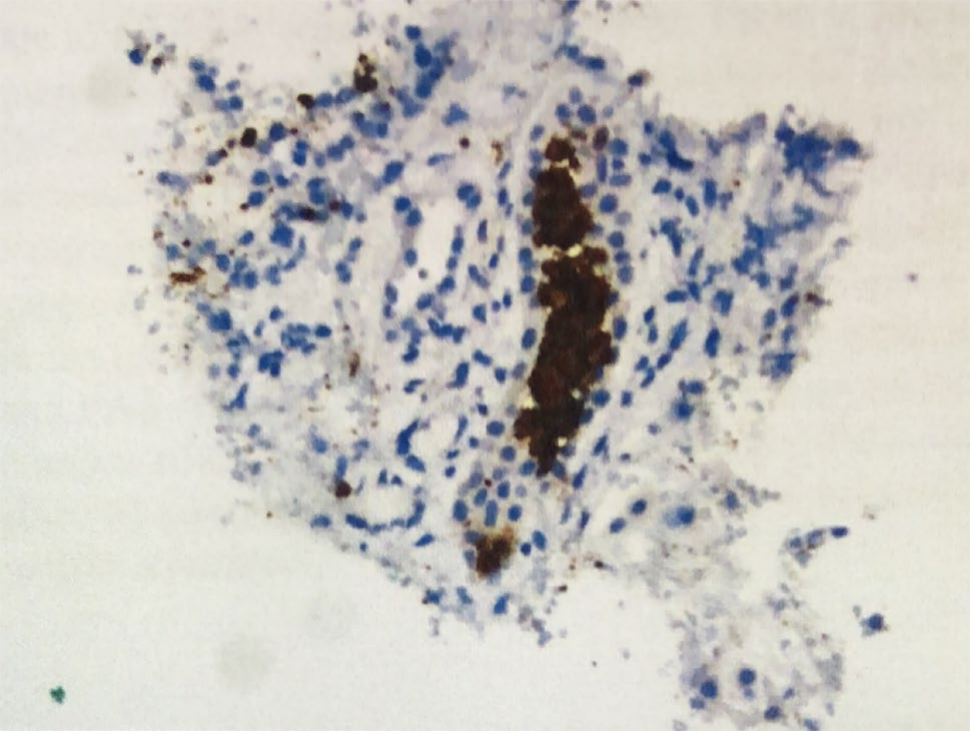
Kidney biopsy showing positive myoglobin stain in the tubular casts under light microscopy

**Fig. 4:**
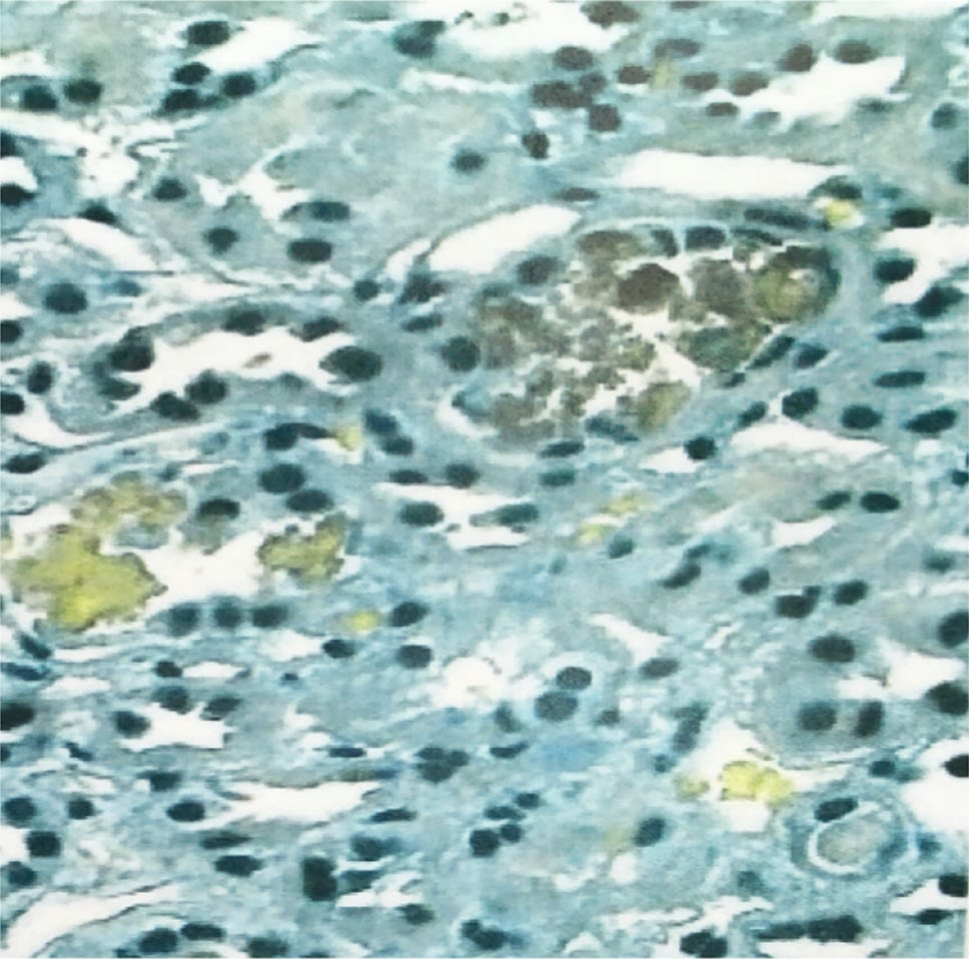
Kidney biopsy showing brownish casts under light microscopy

Over the next week, with continued hemodialysis and steroid treatment, her laboratory and clinical picture improved. CPK levels dropped to within normal range, and creatinine and BUN levels remained consistent and improved (though not normalized) with hemodialysis every other day. Due to persistent oliguria, a renal biopsy was ordered to determine if rhabdomyolysis, SLE, or both were to blame. The biopsy showed muddy brown casts with permanent tubular atrophy, interstitial fibrosis, and massive amounts of myoglobin, implicating rhabdomyolysis, as shown in Figs. 3 and 4, as the main culprit of the acute kidney injury.

## Diagnosis

This patient met 7 out of 11 ARC SLE criteria assigning a diagnosis of Systemic Lupus Erythematosus. The malar and subcutaneous rash, arthritis, leukopenia, Lupus headache, myositis, low C3 levels, and urinary casts confer an SELENA–SLEDAI score of 25, indicating a significant disease activity, as well. We speculate that her severe acute gastrointestinal illness triggered a systemic auto-immune reaction and likely indolent SLE, which ultimately led to severe muscle breakdown and resultant acute kidney failure. Over the next few weeks of her inpatient stay, the rash, along with CPK levels, and urine production, responded well to high-dose corticosteroids, hydroxychloroquine, and hemodialysis allowing for renal recovery. Once stabilized and clinically improved, she was able to be discharged. Unfortunately, she has not followed up with nephrology or rheumatology since discharge and the amount of permanent renal damage remains unknown.

## Discussion

There are various auto-immune disorders with overlapping symptoms that are often difficult to distinguish without thorough workup and biopsies. In this case, mixed connective tissue disease cannot be fully ruled out due to the lack of muscle biopsy and further results, but the diagnosis of SLE seems most likely given the symptoms and reported lab values. While the diagnosis of SLE explained some of our patient’s symptoms and lab results, it did not fully explain the massively elevated CPK levels. We initially contributed the muscle aches to fatigue and dehydration from vomiting and diarrhea, but the grossly elevated CPK level in the 300,000 s suggested rhabdomyolysis or a necrotizing pathology. Rhabdomyolysis causes are divided into three main categories: traumatic, non-traumatic exertional, and non-traumatic non-exertional. Since our patient did not have a history of trauma or exertion, we will focus on discussing the non-traumatic non-exertional causes.

Non-traumatic non-exertional causes of rhabdomyolysis most commonly include drugs, toxins, and infections. Numerous drugs, both medicinal and illicit, have reportedly caused rhabdomyolysis. These include statins, colchicine, cyclosporine, gemfibrozil, protease inhibitors, creatine, caffeine, heroin, cocaine, amphetamines, methadone, and LSD [1]. Furthermore, toxins such as carbon monoxide, snake and insect venoms, or toxins produced by various mushrooms and fish have been implicated in many cases of rhabdomyolysis [2]. While the mechanism is not yet completely understood, both bacterial and viral infections have also been associated with rhabdomyolysis [3, 4]. Offending agents include influenza A and B, coxsackievirus, EBV, herpes simplex, parainfluenza, adenovirus, echovirus, HIV, CMV, *Mycoplasma pneumoniae*, *Legionella*, *Streptococcus*, *Salmonella*, *E. Coli*, *Coxiella*, and *Staphylococcus aureus* [3, 4]. Rhabdomyolysis has also been attributed to endocrine disorders, including diabetes and hypothyroidism, as well as inflammatory diseases, such as polymyositis and dermatomyositis [5, 6].

Due to lack of other contributing factors, we assert that our patient’s rhabdomyolysis was potentially a complication of an acute viral or bacterial gastroenteritis and likely undiagnosed indolent SLE. The previous case reports have connected SLE to an inflammatory auto-immune necrotizing myopathy [7]. Others have implicated SLE in the development of rhabdomyolysis in some patients [8]. These reported cases are extremely rare, and their reported CPK values only reached around 50,000, which makes our particular case even more unusual. Regardless, this atypical presentation may suggest a link between SLE and rhabdomyolysis that is not yet well described in the current literature. In addition to this rarely described connection between rhabdomyolysis and SLE, others have reported an association between rhabdomyolysis and Sjogren’s syndrome [9–11]. However, the current literature does not describe the incidence of rhabdomyolysis secondary to or concurrent with rheumatic diseases.

## Conclusion

SLE spans a wide spectrum of disease presentations with multi-organ involvement which can cause rapid organ failure. Despite establishing the diagnosis of SLE early in the admission, the patient still deteriorated due to complications of her rhabdomyolysis which required a more aggressive treatment plan. As our case and others like it demonstrate, rhabdomyolysis can be seen in patients with SLE, though rarely to this extent [7, 8]. This case report aims to increase physicians’ awareness of rhabdomyolysis as an unusual but possible complication of SLE to aid in more prompt diagnosis and treatment, leading to better patient outcomes.
